# Research interests of family physicians applying for research training

**DOI:** 10.1186/s12909-023-04562-0

**Published:** 2023-08-29

**Authors:** Jennifer K. Carroll, Christina M. Hester, Cory B. Lutgen, Elisabeth Callen, Sharon Hunt, Angela M. Lanigan, Gillian Bartlett-Esquilant, Gretchen Irwin, Warren A. Jones, Natalia Loskutova, Natabhona M. Mabachi, Kolawole S. Okuyemi, Lars E. Peterson, Richard Edward Smith, Cheri Tabel, Amanda Weidner

**Affiliations:** 1 Department of Family Medicine, University of Colorado, 12631 East 17th Ave Box F496, Aurora, CO 80045 USA; 2 American Academy of Family Physicians, Leawood, KS USA; 3 Family & Community Medicine, University of Missouri, Columbia, MO USA; 4 University of Kansas School of Medicine, Wichita, KS USA; 5 University of Mississippi School of Medicine, Jackson, MS USA; 6 The Jones Group of Mississippi, Jackson, MS USA; 7 University of Kansas Medical Center, Kansas City, KS USA; 8 Department of Family and Preventive Medicine, University of Utah School of Medicine, Salt Lake City, UT USA; 9 American Board of Family Medicine, Lexington, KY USA; 10 Pennsylvania State University, Hershey, PA USA; 11 Association of Departments of Family Medicine, Leawood, KS USA

**Keywords:** Research, Family physicians

## Abstract

**Background:**

There is an ongoing need for research to support the practice of high quality family medicine. The Family Medicine Discovers Rapid Cycle Scientific Discovery and Innovation (FMD RapSDI) program is designed to build capacity for family medicine scientific discovery and innovation in the United States. Our objective was to describe the applicants and research questions submitted to the RapSDI program in 2019 and 2020.

**Methods:**

Descriptive analysis for applicant characteristics and rapid qualitative analysis using principles of grounded theory and content analysis to examine the research questions and associated themes. We examined differences by year of application submission and the applicant’s career stage.

**Results:**

Sixty-five family physicians submitted 70 applications to the RapSDI program; 45 in 2019 and 25 in 2020. 41% of applicants were in practice for five years or less (n = 27), 18% (n = 12) were in in practice 6–10 years, and 40% (n = 26) were ≥ 11 years in practice. With significant diversity in questions, the most common themes were studies of new innovations (n = 20, 28%), interventions to reduce cost (n = 20, 28%), improving screening or diagnosis (n = 19, 27%), ways to address mental or behavioral health (n = 18, 26%), and improving care for vulnerable populations (n = 18, 26%).

**Conclusion:**

Applicants proposed a range of research questions and described why family medicine is optimally suited to address the questions. Applicants had a desire to develop knowledge to help other family physicians, their patients, and their communities. Findings from this study can help inform other family medicine research capacity building initiatives.

## Background

Capacity-building for family medicine research has expanded in recent decades [1–5]. Alongside these important achievements, family medicine still faces significant challenges to the engagement of family physicians in research participation and in supporting their development into investigators. The pool of family physicians with research and mentorship experience remains small. In 2016, only 34% of family medicine department chairs reported their department had a high research capacity [6]. Less than a third (31.5%, or 50 out of 160 departments of family medicine) had NIH awards in 2021, and the *total* dollars from the NIH to family medicine departments was about twice the mean of *one* internal medicine department in 2021 [7].

Barriers to family physicians engaging in research include an emphasis on clinical activities, high levels of burnout,[8, 9] the perception of a lack in training or skills to “do research,” inadequate resources, lack of access to mentoring, and, although research is viewed as a high priority for the specialty, it is secondary to clinical duties and requires infrastructure not readily available to family physicians in many settings [10, 11]. Yet, we also know that family physicians are curious – questions that occur in the course of clinical work are very common [12]. Family physicians are also well-suited to engage in research, as they address a myriad of issues, are trained in patient- and community-centered approaches to care, and often serve as a bridge between the clinic and community. Engagement in research is greatest if the problem is viewed as relevant to improving care and the methods are feasible to deploy in a family physician’s practice setting. To date, a more in-depth examination of what these questions and research approaches might be has not been completed. Knowing this information may better inform capacity building initiatives in family medicine research. In this paper, we describe the research questions, themes, and study populations of interest to family physicians acquired through a capacity building initiative. This capacity building program represents a new approach for research training for family physicians, who represent a large sector of the healthcare workforce in the US, to attempt to help address the challenges to research capacity building in the field. Findings may also be broadly relevant to other clinical domains seeking to cultivate research and scholarship among busy “real-world” clinicians across the spectrum of career stages.

## Methods

**Data source**. In 2019, the American Academy of Family Physicians (AAFP) Foundation and the AAFP National Research Network partnered to launch the Family Medicine Discovers Rapid Cycle Scientific Discovery and Innovation (FMD RapSDI) program, the Foundation’s new Scientific Signature Program specifically designed to provide a venue for family physicians to explore clinically inspired questions in a mentored research program. FMD RapSDI is intended to facilitate research engagement and leadership development among family physicians. The FMD RapSDI program funds two family physicians each year to undertake a mentored research project as FMD RapSDI Scholars. The program supports short-term, innovative, high-impact projects, and no previous research experience is required.

**Study design**. We used a Rapid Assessment Procedures qualitative analysis, incorporating a matrix approach [13–15] informed by principles of grounded theory, and as part of the grounded theory methods, incorporated content analysis for the inductive process [13, 16] to analyze the FMD RapSDI applications from 2019 to 2020. Rapid qualitative analysis techniques have been used previously in health services research and related fields [16–19]. We report our methods here in accordance with the **CO**nsolidated Criteria for **RE**porting **Q**ualitative Research checklist (COREQ) criteria [20]. As the COREQ criteria were developed for qualitative studies using focus groups and individual interviews, we adapted our reporting of the COREQ criteria as appropriate for our data source. This study received IRB approval as an exempt study under Category 4.

**Participant sampling strategy**. We analyzed all applications submitted to the FMD RapSDI program in 2019 (n = 45) and 2020 (n = 25). For the applicants who submitted applications in both years (n = 6), as their topics were either new or substantially revised, they were counted twice–i.e., analyzed in each year. The data used for the qualitative analysis consisted of applicants’ responses in the application fields to the questions shown in Table 1.

**Table 1:** Data collected from applications

Data	Question (If Applicable)
Significance to applicant Character limit: 3000	Please describe why your clinic-inspired question is of particular interest to you.
Significance to family medicine Character limit: 3000	How do you believe other family physicians, their patients, and the discipline of family medicine may benefit from the answer to your proposed question?
State/Region	
Employer	
Number of years in practice	

We conducted Internet searches of each applicant’s employer to classify them into AAFP employer categories (e.g., self-employed, physician group, university-owned, private for-profit system, private non-profit system).

**Analysis**. The primary analysis team consisted of six individuals with experience in qualitative research. The team identified the key domains for further analysis: nature of topics and questions, significance to applicant, and significance to family medicine. Key words and phrases were abstracted by the analysis team by choosing words or phrases that captured the essence of the application’s core content.

The team prepared summary templates of the qualitative data, then discussed initial observations: emerging themes, degree of similarity versus difference, observations about study populations and/or settings, and unexpected findings or other reflections. These observations were noted in the summary templates.

Next, the team selected applicant characteristics to guide the matrix analysis. We chose number of years in practice for two reasons: (1) we believed it would provide an interesting lens through which to explore themes and interpretation of the domains, and (2) the applicants were distributed in sufficient numbers across the numbers of years of practice, allowing us to analyze the findings using this applicant characteristic. The team also compared applications for similarities and differences between 2019 and 2020, which occurred during the COVID-19 pandemic and marked social and political unrest.

In accordance with published methods for matrix development,[13, 16] the team completed and critically appraised matrices for the applications based upon guiding questions in Table 1. Cross-cutting themes/orientations of the questions emerging from the matrices were identified based on information in each of the three sections (question, significance to applicant, significance to FM). Themes/orientations were recorded for each applicant’s question. The team discussed findings from the matrix analyses and prepared the narrative account of the findings.

Finally, we conducted descriptive statistics to characterize our applicant sample in aggregate for both years.

**Data saturation**. Data saturation was achieved for the significance to applicant and significance to family medicine domains, but not for the research question domain. Saturation was verified in two ways. First, we obtained verbal consensus from the analysis team that we had reached redundancy in the findings where applicable. Second, two members of the analysis team reviewed the matrix analysis materials which also indicated redundancy of themes across the domains noted previously.

## Results

The FMD RapSDI program received 70 applications from 65 family physicians in 2019 (n = 45) and 2020 (n = 25). Six applicants submitted applications for both years, either as new or revised applications. Three applicants submitted more than one unique application in 2019; no applicants submitted more than one application for 2020. Application and applicant numbers are shown by year and career stage in Table 2.

**Table 2:** Number of Applicants and Applications for the RapSDI Program

	Number of applications (number of applicants)*		
	2019	2020	**Total**
0–5 years in practice	18 (17)	10 (10 – includes 2 repeat applicants from 2019)	**28 (27)**
6–10 years in practice	6 (6)	5 (5)	**11 (11)**
11 + years in practice	21 (17)	10 (10 – includes 4 repeat applicants from 2019)	**31 (27)**
**Total**	**45 (40)**	**25 (25)**	**70 (65)**

*in 2019, three applicants submitted more than one application, and 6 applicants submitted applications in both 2019 and 2020. The number in parenthesis reflects number of applicants.

***Applicant characteristics.*** Table 3 shows applicant characteristics for each year and overall. The majority of applicants were either 0–5 years or 11 + years in practice. Applicants represented 24 states, with the South and West regions most commonly represented. 49% of applicants had a university affiliation; 52% were faculty in a residency program; and 25% were self-employed. Using available AAFP data, the RapSDI applicant pool is broadly representative of family physicians as a whole,[21] with the exception of a few slight differences. The RapSDI applicant pool has (1) a slightly larger proportion of individuals in an early career phase, (2) a higher representation of university affiliated individuals, and (3) a slightly higher percentage of individuals employed in a federally qualified health center setting.

**Table 3:** RapSDI Applicant Characteristics

Characteristic	2019	2020	Combined (2019 and 2020)
Applicants (total #)	40	25	65
Proposals (total #)	45	25	70
**Years in Practice**			
Average Years in practice	9.5	12.7	10.7
Median Years in practice	8.5	8	8
Years in practice (0–5)	43% (n = 17)	40% (n = 10)	41% (n = 27)
Years in practice (6–10)	18% (n = 7)	20% (n = 5)	18% (n = 12)
Years in practice (11+)	40% (n = 16)	40% (n = 10)	40% (n = 26)
**% with University affiliation**	48% (n = 19)	52% (n = 13)	49% (n = 32)
Total # of states*	20	15	24
**Geographic Regions**			
% Northeast	23% (n = 9)	24% (n = 6)	23% (n = 15)
% South	30% (n = 12)	40% (n = 10)	34% (n = 22)
% Midwest	23% (n = 9)	4% (n = 1)	15% (n = 10)
% West	25% (n = 10)	32% (n = 8)	28% (n = 18)
**Employer type (AAFP categories)**			
You (self-employed, majority practice owner, independent contractor, etc.)	30% (n = 12)	16% (n = 4)	25% (n = 16)
Physicians group (single- or multi-specialty)	3% (n = 1)	0% (n = 0)	2% (n = 1)
University-owned (public or private) clinic or hospital	28% (n = 11)	36% (n = 9)	31% (n = 20)
Private for-profit hospital or health system	0% (n = 0)	0% (n = 0)	0% (n = 0)
Private non-profit hospital or health system	25% (n = 10)	12% (n = 3)	20% (n = 13)
Managed care organization or insurance company	0% (n = 0)	0% (n = 0)	0% (n = 0)
Federal, state, or local government, community board, etc. (not including universities)	15% (n = 6)	0% (n = 0)	9% (n = 6)
Locum tenens group/staffing organization	0% (n = 0)	0% (n = 0)	0% (n = 0)
Other	13% (n = 5)	26% (n = 9)	22% (n = 14)
**% Residency program**	48% (n = 19)	60% (n = 15)	52% (n = 34)
**% FQHC or FQHC lookalike**	18% (n = 7)	28% (n = 7)	22% (n = 14)

*Each value shows the number of unique states per year, and the total number of unique states (for both years)

***Nature of the topics and questions.*** There was a wide range of topics of interest; 144 unique key words or phrases were identified. The most common key words or phrases were studies of new technologies or tools for primary care (n = 20, 28%), interventions or approaches to reduce cost or be cost effective (n = 20, 28%), studies to improve screening or diagnosis (n = 19, 27%), studies addressing mental, emotional or behavioral health (n = 18, 26%), and studies to improve care for vulnerable or disadvantaged populations (n = 18, 26%). The key words represented a variety of topics: disease-specific, health promotion or disease prevention, process of care, improving communication and the patient-physician relationship, social determinants of health and health disparities.

**Fig. 1:**
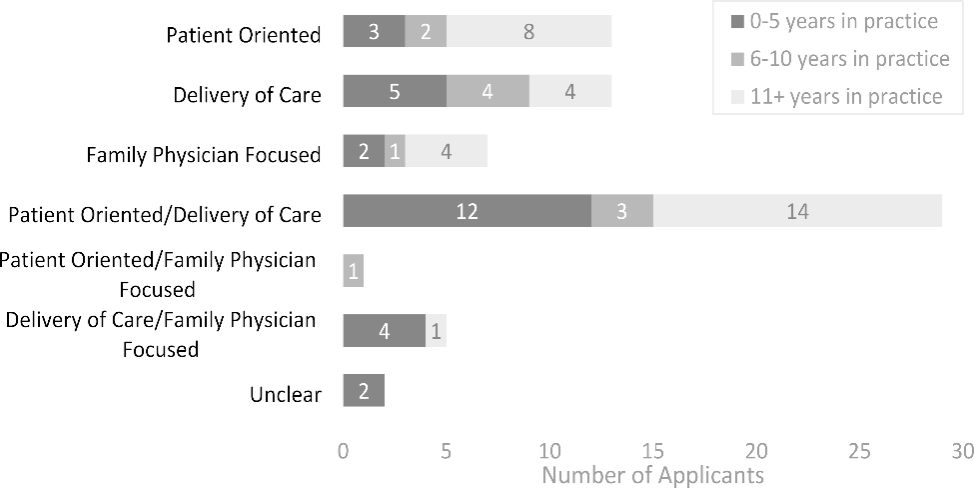
The distribution of themes among research topics across applicants’ career stages

Despite the heterogeneity in topics, there were similarities regardless of applicant career stage and/or year of application. Four themes/orientations were identified across the applications: orientation to patient outcomes; orientation to optimization of delivery of care; family physicians as the population of interest; orientation to community; and/or some combination of the above. The distribution of themes across career stage is shown in Fig. 1. For example, most questions aimed to understand an association either between patient- and disease-related characteristics, or between an intervention and outcomes. Most questions also explicitly involved patient-related outcomes in a population of the applicant’s clinic and/or their own patient panel, a theme also seen across all career stages.

There were some differences in the topics by year with applications in 2020 including COVID-19, social justice, and racism, topics not seen in the 2019 applications. There was further emphasis on questions related to one or more social determinants of health and telemedicine for 2020.

***Significance to applicant.*** Most applicants, regardless of number of years in practice, stated that the significance to them was that their question/topic represented an issue they faced with patients. They cited high motivation to help their patients and to develop an intervention to improve patient and/or practice outcomes. A priority for many applicants was to keep their patients engaged in their own care and in their medical home; however, a few explored interprofessional or cross-specialty collaborations. Some cited personal or patient experiences with the problem or condition while others had a focus on potential national impact; for example by testing a new technology, tool, or approach to screening that could be applicable to patients seen in a broad variety of family medicine settings.

Although there were more similarities than differences across years in practice for the significance to the applicant, there were some differences. Applicants in practice for five years or less tended to describe the significance to them to address perceived knowledge or training gaps. Applicants in practice 6–10 years tended to focus on an intervention to change care delivery and/or patient education in the applicant’s practice to improve patient outcomes. Applicants in practice 11 + years tended to frame their research topic to address a problem within their own clinical setting, usually an issue that the applicant considered vexing or of high priority across their career/foundation of previous experience.

***Significance to family medicine.*** Broader significance to the field of family medicine was often stated in terms of the belief that family medicine is the specialty best suited to identify and address the applicant’s question or topic of interest.

Applicants often viewed their topic’s significance to family medicine as a means to meet patients’ needs and improve outcomes, access to care, prevention, address specific pain points related to the care of individuals with one or more chronic conditions, build specific skills or content expertise, and (for 2020 applications) address telemedicine and virtual group visits for patients. Other applicants stated that the significance of their questions was grounded in the potential to improve implementation of practice guidelines and discover new ways to promote team-based and comprehensive care.

Family medicine as foundational to patients’ health, having a person-and community-centered orientation, and embodying a broad scope of practice was emphasized as a guiding principle.

Applicants considered a variety of complex issues as relevant for family physicians’ practices–for example, gun violence, learning how to use and interpret several forms of practice data, leading inter-professional collaborations, and studying integration of services on patient and practice outcomes.

## Discussion

Analysis of the FMD RapSDI applicant dataset revealed a breadth of family physicians, representing a diverse mix of geographic locations, employer types, and career stages. About half of the applicant pool represented family physicians working in residency programs, a group not as commonly represented among efforts to build research capacity pipeline in family medicine [22]. The high percentage of applicants from residency programs was an unexpected finding, as FMD RapSDI was designed to reach family physicians outside of academic-based settings – where opportunities to participate in research may be incorrectly presumed to be more prevalent. Interest in RapSDI among residency faculty may indicate that segmentation of family physicians with academic affiliations may be important in determining how best to support research capacity building efforts among family physician faculty in different contexts (e.g., residency programs located in community-based versus academic-based settings). We were unable to distinguish between applicants from residencies that are community-based and academic-based with the data collected here beyond what is presented in Table 3.

We observed a large variety of questions and topics and a range of complexity, similar to another description of a research capacity building initiative [23]. There was, overall, more similarity than difference among the applicant pool for the significance that applicants described for themselves personally and the broader specialty of family medicine. Broad topical differences between 2019 and 2020 were that 2020 applications had questions related to social justice, racism, and COVID (all new) and a greater proportion of applications focused on social determinants of health and telemedicine compared to 2019. Four themes/orientations were identified across the applications: orientation to patient outcomes, orientation to optimization of delivery of care, family physicians as the population of interest, orientation to community, and some combination of the above.

Our findings reflect and expand on previous efforts to build a culture of inquiry, models to advance scholarship and interest in family medicine research via pragmatic approaches [23, 24]. A study conducted by the Family Practice Inquiries Network reported some similar findings, such as the importance of studying effective treatments and screening approaches [1]. Another study created taxonomies to categorize questions. Some of the most common question categories aligned with our findings (such as “How should I manage disease or condition x?”); whereas others did not (“What is the dose of drug x?”) [12].

Many applications either implicitly or explicitly involved an aspect of participatory research or embodied assumptions about the value of it as a foundational principle. This aligns with the reality that family physicians’ questions derive from their established relationships with their patients, patient-centered skills, and training. Family physicians are accustomed to having some degree of comfort, or at least curiosity, about uncertainty; favor pragmatism; and combine patient care and research, which provides a unique “grounding” perspective [25]. Findings from this analysis speak to the importance of amplifying the volume of consciously engaged family physicians in research, providing a means to have family physicians set the research agenda themselves directly, and the unique position (and duty) of family physicians to conduct research [26].

The level of interest in core topic areas, breadth of questions asked, and reasons for significance for the applicants and for the discipline of family medicine signals the need to consider additional support, partnerships, and opportunities to cultivate family medicine research capacity. While many applicants acknowledged significant historical barriers to their participation in research similar to previously published reports,[12, 27, 28] an over-arching theme that seemed to ignite these applicants’ interest in research was the desire to help create new knowledge, which has been cited as a reason family physicians choose to participate in practice-based research networks [29]. In our study, family physicians’ questions embodied two core tenets of research in family medicine: [1] the need to build the knowledge base for diagnosis and management for common conditions in primary care and [2] research on how best to deliver patient care [30].

This study’s findings should be interpreted in the context of its limitations. We had limited qualitative data and other data sources for applicant descriptive characteristics. Due to the nature of the application process, we did not incorporate other types of qualitative data (such as individual interviews or focus groups) with the narrative data used (the applications). We unfortunately lacked data that we did not capture --e.g., gender and race/ethnicity of applicant. By design, the application questions and responses were intended to be brief, which may have limited our ability to do in-depth interpretation for some of the issues we explored in the analysis. Finally, this analysis covers only two years and a limited number of family physicians relative to the entire specialty. While generalizability is an issue, our work provides some important preliminary insights.

**Future directions.** Findings from this study are informing programmatic development for the FMD RapSDI program as well as research capacity building programs in family medicine generally. At the programmatic level, the findings help us consider ways to target applicant groups potentially under-represented, such as physicians who are unaffiliated with a residency program or department of family medicine. From a diversity, equity, and inclusion perspective, we will collect gender, race and ethnicity information in order to track and optimize equity and representativeness of the applicant pool.

Understanding what piques the curiosity of practicing family physicians and drives them to apply for a mentored research opportunity is useful for those seeking to engage family physicians in research as well as those looking to ensure that research is responsive to the needs of practicing family physicians. Importantly, the applicants to RapSDI were not all early career: 40% of applicants each cycle had 11 + years’ experience post-residency. Understanding that family physicians at all stages are interested in proposing, conducting, and participating in research is important for informing those developing and implementing research capacity building initiatives and those funding training awards and programs with an expanded perspective on who to target for program involvement.

To inform family medicine research capacity building efforts broadly, future work should consider new strategies-e.g., for mentorship, coaching, and/or funding- to support and sustain family physicians’ continued engagement in research, highlighting the spectrum of what “doing research” means; and develop strategies to amplify the relevance of family physicians’ research questions and the potential impact of answering them to potential research collaborators, funders, and sponsors.

## Conclusion

Among a national sample of family physicians applying to a mentored research program, a wide range of research topics and questions was observed. The most common topics were studies of new technologies or tools for primary care, interventions or approaches to reduce cost or be cost effective, to improve care for vulnerable or disadvantaged populations, and to improve screening or diagnosis, addressing mental, emotional or behavioral health. Common themes related to the significance of the questions for the applicants and the field of family medicine were the belief that family medicine is the specialty best suited to identify and address the applicant’s question or topic of interest, that results could help improve knowledge gaps for physicians/clinicians, patient outcomes, or patient engagement in care.

## Data Availability

All datasets generated and/or analyzed during this study are not publicly available as the data originated from individual deidentified applications to the FMD RapSDI program for the 2019 and 2020 program cycles. The applications are the intellectual property of the individual applicants and the FMD RapSDI program. Data generated from analysis of the applications and the applicant characteristics are included in this published article or can be requested from the corresponding author and discussed on a case-to-case basis.

## List of abbreviations

AAFP: American Academy of Family Physicians
FMD RapSDI: Family Medicine Discovers Rapid Cycle Scientific Discovery and Innovation
COREQ: Consolidated criteria for reporting qualitative research

## References

[1] Lindbloom EJ, Ewigman BG, Hickner JM (2004). Practice-based research networks: the laboratories of primary care research. Med Care.

[2] North American Primary Care Research Group (NAPCRG). Grant Generating Project (GGP) https://www.napcrg.org/programs/grantgeneratingproject-ggp/about-ggp/. Accessed 15 March 2021.

[3] North American Primary Care Research Group (NAPCRG). Building Research Capacity (BRC) Program https://www.napcrg.org/programs/buildingresearchcapacity-brc/building-research-capacity-brc-program/. Accessed 15 March 2021.

[4] Association of Departments of Family Medicine. The Family Medicine Physician Scientist Pathway (PSP) Program https://www.adfm.org/programs/physician-scientist-pathway/. Accessed 15 March 2021.

[5] American Academy of Family Physicians Foundation, Grants, Awards. https://www.aafpfoundation.org/grants-awards.html. Accessed 15 March 2021.

[6] Weidner A, Peterson LE, Mainous AG 3rd, Datta A, Ewigman B. The current state of Research Capacity in US Family Medicine Departments. Fam Med. 2019;51:112–9. 10.22454/FamMed.2019.180310 30736036

[7] Blue Ridge Institute for Medical Research. Ranking Tables of NIH Funding to US Medical Schools in 2020 as compiled by Robert Roskoski Jr. and Tristram G. Parslow http://www.brimr.org/NIH_Awards/2020/default.htm. Accessed 30 March 2023.

[8] Eden AR, Jabbarpour Y, Morgan ZJ, Wilkinson E, Peterson LE (2020). Burnout among Family Physicians by gender and age. J Am Board Fam Med.

[9] Ofei-Dodoo S, Loo-Gross C, Kellerman R, Burnout (2021). Depression, anxiety, and stress among Family Physicians in Kansas responding to the COVID-19 pandemic. J Am Board Fam Med.

[10] Liaw W, Eden A, Coffman M, Nagaraj M, Bazemore A. Factors Associated with successful Research Departments A qualitative analysis of Family Medicine Research Bright Spots. Fam Med.51:87–102. 10.22454/FamMed.2018.652014 30376674

[11] Brocato JJ, Mavis B (2005). The research productivity of faculty in family medicine departments at U.S. medical schools: a national study. Acad Med.

[12] Ely JW, Osheroff JA, Ebell MH, Bergus GR, Levy BT, Chambliss ML (1999). Analysis of questions asked by family doctors regarding patient care. BMJ.

[13] Averill JB (2002). Matrix analysis as a complementary analytic strategy in qualitative inquiry. Qual Health Res.

[14] Miles MB, Huberman AM (1994). Qualitative data analysis: an expanded sourcebook.

[15] Marsh GW (1990). Refining an emergent life-style-change theory through matrix analysis. ANS Adv Nurs Sci.

[16] Beebe J (2001). Rapid assessment process: an introduction.

[17] Burgess-Allen J, Owen-Smith V (2010). Using mind mapping techniques for rapid qualitative data analysis in public participation processes. Health Expect.

[18] Neal JW, Neal ZP, VanDyke E, Kornbluh M (2015). Expediting the analysis of qualitative data in evaluation:a Procedure for the Rapid Identification of Themes from Audio Recordings (RITA). Am J Eval.

[19] Taylor B, Henshall C, Kenyon S, Litchfield I, Greenfield S (2018). Can rapid approaches to qualitative analysis deliver timely, valid findings to clinical leaders? A mixed methods study comparing rapid and thematic analysis. BMJ Open.

[20] Tong A, Sainsbury P, Craig J (2007). Consolidated criteria for reporting qualitative research (COREQ): a 32-item checklist for interviews and focus groups. Int J Qual Health Care.

[21] American Academy of Family Physicians. Facts About Family Medicine https://www.aafp.org/about/dive-into-family-medicine/family-medicine-facts.html Accessed 11 May 2023.

[22] Mullen R, Weidner A, Liaw W, Mainous AG, Hester CM, Goodyear-Smith F (2020). Family medicine research capacity in the USA. Fam Pract.

[23] Lawson PJ, Smith S, Mason MJ, Zyzanski SJ, Stange KC, Werner JJ (2014). Creating a culture of inquiry in family medicine. Fam Med.

[24] Nutting PA, Stange KC, Rakel RE (2001). Practice-based research: the opportunity to create a learning discipline. Textbook of family medicine.

[25] Macaulay AC (2007). Promoting participatory research by family physicians. Ann Fam Med.

[26] Del Mar C, Askew D (2004). Building Family/General Practice Research Capacity. The Annals of Family Medicine.

[27] Mainous AG 3. Culture, resources, or a bit of both: increasing scholarship in family medicine. Fam Med. 2014;46:501–2. 25058541

[28] Beckett M, Quiter E, Ryan G, Berrebi C, Taylor S, Cho M (2011). Bridging the gap between basic science and clinical practice: the role of organizations in addressing clinician barriers. Implement Sci.

[29] Yawn BP, Dietrich A, Graham D, Bertram S, Kurland M, Madison S (2014). Preventing the voltage drop: keeping practice-based research network (PBRN) practices engaged in studies. J Am Board Fam Med.

[30] Mainous AG 3rd, Hueston WJ, Ye X, Bazell C. A comparison of family medicine research in research intense and less intense institutions. Arch Fam Med. 2000;9:1100–4. 10.1001/archfami.9.10.1100 11115214

